# Phytobezoar: An unusual cause of small bowel obstruction

**DOI:** 10.1016/j.amsu.2021.01.048

**Published:** 2021-01-25

**Authors:** Mounir Bouali, Ahmed Ballati, Abdelilah El Bakouri, Khalid Elhattabi, Fatimazahra Bensardi, Abdelaziz Fadil

**Affiliations:** a Department of General Surgery, University Hospital Centre Ibn Rochd, Casablanca, Morocco; b Faculty of Medecine and Pharmacy, Hassan II University, Casablanca, Morocco

**Keywords:** Gastric surgery, Phytobezoar, Small bowel obstruction, Acute surgical abdomen

## Abstract

Phytobezoars are concretions of indigested fruit and vegetables fibers in the gastrointestinal tract. The past of gastric surgery is most common risk factor of phytobezoar. We present the case of a 39-year-old female was admitted to the emergency department and who presented with small bowel obstruction due to phytobezoar, her past medical history was marqued by truncal vagotomy and simple suture recurrent perforated gastric ulcer 15 years earlier. Her postoperative recovery was uneventful.

## Highlights

• The phytobezoar are concretion on undigested fruits and vegetables fibers in the gastrointestinal tract.
• The occurrence of small bowel obstruction caused by phytobezoar is extremely rare.
• The past of gastric surgery is most commun factor of phytobezoar.

## 1 Introduction

Bezoar is an uncommon condition that results from the accumulation of a variety of solid masses and substances of different types and shapes. It is an entirely natural process in the stomach but we can found it in the digestive tract. Several types of bezoars can be distinguished according to the substance ingested:The lacto-bezoard, observed in infants, the phytobezoard, formed by a conglomerate of vegetable fibers, other various intragastric substances can constitute bezoars including fungal conglomerates, food residues, and the trichobezoar, which constitutes 55% of all bezoars, is constituted of hairs, bristles or carpet fibers. It is known as “Rapunzel syndrome” [1]. In our clinical case, we will discuss the phytobezoar causing small bowel obstruction who had surgery for an obstructing small bowel phytobezoar.

This case is reported in line with the SCARE criteria [2].

## 2 Case presentation

**Fig. 1:**
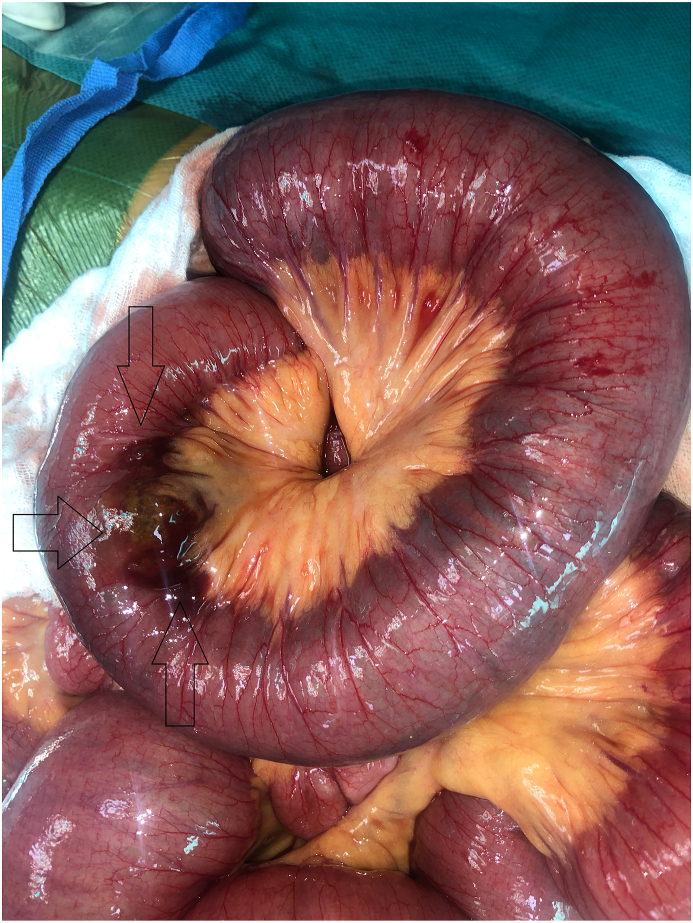
Obstruction by a phytobezoar in the distal ileum.

**Fig. 2:**
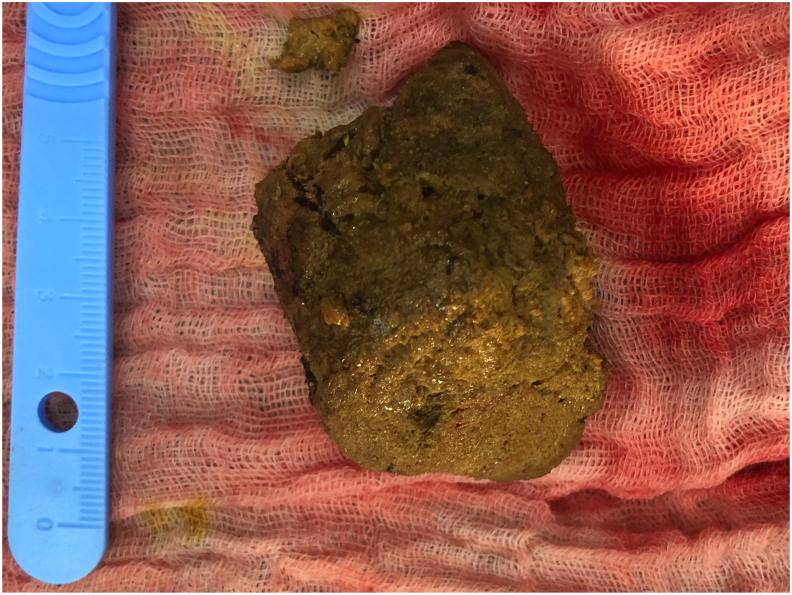
Extracted phytobezoar.

39-year-old female patient, her past medical history was marqued by truncal vagotomy and simple suture recurrent perforated gastric ulcer 15 years earlier. The history of the disease went back to one week by the installation of an abdominal pain, bloating, associated with vomiting. On clinical examination, the patient presented a slightly distended abdomen with diffuse tenderness. She had a blood pressure of 130/70 mm Hg, a pulse rate of 88 beats/min, a respiratory rate of 16 breaths/min, and body temperature of 37.3 C. Laboratory evaluation revealed a white blood cell count of 13.7 × 10^4^/L with 80% neutrophils and hemoglobin level of 15.3 g/L. Liver and kidney function tests were normal, An abdominal CT scan was performed, which revealed a diastatic jejunal-graft occlusion on parietal thickening. Emergency laparotomy was performed. During exploration, a hard obstructing bezoar in distal ileum with a perforating lesion (Fig. 1) and another one in the stomach were discovered. A gastrotomy was performed which allowed the extraction of the foreign gastric body bezoar (Fig. 2) and underwent a 3 cm segmental graft resection of small bowel with terminoterminal grelo-grelic anastomosis for the second bezoar. The discharge was on the 4 th postoperative day.

## 3 Discussion

There are several types of bezoars, depending on the substance ingested: trichobezoar, phytobezoar and lactobezoar. Trichobézoar is a rare disease, the female sex is the most affected (90% of cases) and the age of onset is in 80% of cases less than 30 years old, with a peak incidence between 10 and 18 years old. Psychological pathologies are sometimes found such as psychomotor retardation or isolation, but only 9% of children with trichobezoar are reported to have real psychiatric problems. The trichobezoar is most often gastric, but it can extend to the small intestine or even the transverse colon [3]. In our case, the patient had a phytobezoar, which is a compact mass of fibers, seeds, plant roots that collect in the stomach or intestine. Other food particles such as fats and salt residues are incorporated and contribute to their development [4]. Causes of bezoar include gastroparesis, associated with diabetes, chronic kidney disease, history of vagotomy, partial gastrectomy or a previous impairment of digestive motility during systemic scleroderma [5]. Classical promoting factors are, among others, gastric emptying disorders, loss of normal pyloric motor functions, the consequences of partial gastrectomy, and a high-fiber diet. Antisecretory gastric treatments seem to play a role: the hypoacidity that they cause a decrease in the activity of enzymes (pepsin, cellulase) involved in the disintegration of dietary fibers [1]. Poor chewing, for example due to the absence of teeth and tachyphagia can lead to the formation of bezoars. Ingestion of hair or non-digestible material, or excessive consumption of poorly digestible foods can also lead to the formation of bezoars. These are rare events, more common in the small intestine than in the stomach [6]. Clinical presentation in patients with phytobezoars depends on the type, location within the gastrointestinal tract, and presence of predisposing factors. Gastric bezoars can present with epigastric discomfort and nonspecific abdominal pains [7]. Clinical manifestations depend on the location of the bezoar. Gastric bezoars may cause dyspepsia, food intolerance, abdominal pain, vomiting, nausea, pyrexia, gastric outlet obstruction, and bleeding from a gastric ulcer. The clinical symptomatology is varied and non-specific. We can found: Pain, vomiting, anorexia, or weight loss. The clinical examination may note the presence of alopecia, epigastric mass, or pyloric stenosis syndrome [8]. Bezoars can cause gastric ulcers, intestinal obstruction, perforation or hemorrhage. In our case, phytobezoar caused small bowel obstruction. The hemogram may indicate a hypochromic anemia moderate or hypo-albuminemia [9]. Endoscopy is the technique of choice for the diagnosis and categorization of bezoars. Trichobezoars are black, while phytobezoars are polychrome, yellow, brown or green [10]. X-rays of the abdomen may show a mass that invades the gastric airways, but exceptionally they can reconstitute the mass involved in the occlusion: risk of confusing it with faeces or an abscess. CT and MRI scans reveal a mass with non-contrasting air pockets [1]. The performance of an MRI scan is only reported twice in the literature, in particular about a case of complicated bezoar ulcer [11]. The authors insist on the fact that the large size of a trichobezoar can even make it more difficult to identify it by MRI, because its hypo signal overall is not a signal of the sequences used can be easily confused with the hyposignal of intragastric air [12]. In our case, the patient had benefited from an abdominal CT scan that revealed a diastatic graft occlusion on parietal thickening, without etiology of the bezoar. Several therapies have been reported in the literature. Thus, in the presence of small trichobezoards, some authors propose the use of abundant drinking associated with the taking of transit gas pedals. In case of failure, endoscopic extraction can be attempted, using laser beams to fragment it. Extracorporeal lithotripsy has been proposed in literature as an alternative. However, these techniques are often incomplete and expose the patient to a high risk of intestinal occlusion on a trichobezoar fragment [13]. In our case, the patient benefited from the extraction of a “bezoar” foreign body by gastrostomy and 3cm segmental hail resection carrying a preperforative lesion with terminoterminal grelo-grelic anastomosis. The preventive measures are low-fiber diet, more water, proper mastication, and treatment of gastrointestinal motility disorders.

## 4 Conclusion

Small bowel phytobezoar is an uncommon cause of acute intestinal obstruction. Surgery is often required for diagnostic and treatment. The manner diet modification is the best way for prevention of small bowel obstruction due phytobezoard.

## Ethical approval

I declare on my honor that the ethical approval has been exempted by my establishment.

## Sources of funding

The authors declared that this study has received no financial support.

## Author contribution

**Ahmed Ballati**: Corresponding author writing the paper.

**Mounir Bouali**: study concept.

**Abdelilah Elbakouri**: writing the paper.

**Khalid Elhattabi**: correction of the paper.

**Fatimazahra Bensardi**: correction of the paper.

**Abdelaziz Fadil**: correction of the paper.

## Research registration number

Researchregistry5198.

## Guarantor

DOCTEUR AHMED BALLATI.

## Consent

Written informed consent for publication of their clinical details and/or clinical images was obtained from the patient's parents.

## Provenance and peer review

Not commissioned, externally peer-reviewed.

## Declaration of competing interest

The authors report no declarations of interest.
